# From theory into practice: insights from a real-world implementation model for tailored exercise prescription in chronic diseases

**DOI:** 10.1186/s13102-025-01419-5

**Published:** 2025-12-29

**Authors:** Federica Duregon, Giulia Quinto, Marco Vecchiato, Sara Faggian, Nicola Borasio, Veronica Baioccato, Sara Ortolan, Andrea Gasperetti, Andrea Ermolao, Daniel Neunhaeuserer, Francesca Battista

**Affiliations:** 1 Sports and Exercise Medicine Division, Department of Medicine, University of Padova, Padova, Italy; 2 Exercise is Medicine Italy, Padova, Italy

**Keywords:** Adherence, Cardiopulmonary exercise testing, Compliance, Exercise therapy, Implementation

## Abstract

**Background:**

Although physical exercise is an evidence-based treatment for patients with chronic diseases, providing benefits in terms of morbidity, mortality and quality of life, its implementation in real-world healthcare systems is still limited. Even when physicians recommend physical activity, compliance and adherence to exercise programs remain very low. This study aims to implement a real-world model for tailored exercise prescription (TEP) in an outpatient clinic to evaluate feasibility, effectiveness, compliance and adherence.

**Methods:**

A TEP was set for each participant, based on a complete clinical and functional evaluation including cardiopulmonary exercise testing and fitness test battery. Subsequent supervised training sessions (STS) were performed for at least 6 weeks. After 6 months functional evaluations were repeated, also assessing compliance with general recommendations and adherence to the prescribed exercise program.

**Results:**

A total of 312 patients (44% male) with a mean age of 52.1 ± 13.6 years were enrolled. The most frequent main chronic conditions were obesity (47%), solid organ transplantation (32%), primary cardiovascular diseases (8%) and cancer (4%). The initial STS program was completed by 85.9% of patients, all without adverse events. Patient compliance, measured as attendance at the follow-up meeting, was 53.2%, while adherence to the TEP during the 6-month program was 44.9%.

**Conclusion:**

A real-word model for TEP followed by a period of STS is feasible in patients with chronic diseases in a real outpatient clinical setting. However, intervention strategies based on behavioral change and motivation are needed to foster greater compliance and adherence in the mid-to-long term.

**Supplementary Information:**

The online version contains supplementary material available at 10.1186/s13102-025-01419-5.

## Background

Physical exercise is an evidence-based treatment and should be prescribed for patients with various chronic conditions [1]. All physicians should carefully decide on the appropriate exercise prescription for each patient and recommend structured exercise in addition to a physically active lifestyle [2]. Some patients with specific chronic conditions or those severely deconditioned need more in-depth evaluations to better adapt the tailored exercise prescription (TEP) [3]. In these cases, assessing cardiorespiratory fitness and efficiency through cardiopulmonary exercise testing (CPET) and fitness tests is recommended to detect functional limitations or contraindications to different exercise types, frequencies or intensities, thereby improving the accuracy of structured and individualized TEP [4, 5]. Fitness and exercise capacity are valid and reliable measures of training status, as well as useful health indicators and prognostic markers, and should be regularly measured in patients with chronic conditions [6, 7].

Despite the demonstrated impact of exercise on the most prevalent chronic diseases, significant barrier and implementation problems regarding patients’ compliance and adherence to regular physical activity (PA) persist in the real world, particularly in healthcare settings [8]. Additionally, it remains unclear whether exercise in supervised settings leads to a long-term increase in physical activity compliance and adherence in unsupervised environments [9].

Compliance has been defined by the World Health Organization as “the extent to which the patient’s behavior matches the prescriber’s recommendations”, while adherence is defined as “the extent to which a person’s behavior, taking medication, following a diet, and/or executing lifestyle changes, corresponds with agreed recommendations from a health care provider” [10–12]. Therefore, while compliance is a “passive” action, simply like “doing as told” and returning to the outpatient clinic for control, adherence is a proactive behavior leading to lifestyle change [8, 13]. It is estimated that more than a quarter of the world’s adult population (1.4 billion) does not adhere to PA recommendations [14]. In high-income countries, 26% of men and 35% of women are insufficiently physically active, compared to 12% of men and 24% of women in low-income countries [15]. These figures align with Italian records, indicating that about one-third of the population (28.1%) is completely inactive [16]. Consequently, the prevalence of insufficiently active individuals is rather high, contributing to the physical inactivity pandemic, that is considered a major global health problem and the fourth leading cause of death worldwide [17, 18].

Various strategies to support and manage exercise therapy in chronic conditions have been described, analyzing risks versus benefits, determining the costs of inactivity, or discussing approaches to prescribe exercise as medicine in general practice [2, 18–20]. However, to the best of authors’ knowledge, few implementation models have included a supervised exercise starting program with Exercise Professionals in a real-word setting to educate patients and increase not only initial compliance, but also long-term adherence to the exercise prescription [14]. Relevant pioneering efforts include the New Zealand "Green Prescription" model, one of the earliest exercise referral schemes involving written prescriptions and support from exercise facilitators [21]. Another notable example is the Swedish physical activity on prescription approach, which has demonstrated increased physical activity levels through individualized exercise counseling within primary care [22]. Additionally, the European Federation of Sports Medicine Associations has proposed a 10-pillar model providing guidelines for promoting physical activity and motivating behavioral changes through strategies like social support and enjoyable activities [23].

Building upon these paradigms, this study aims to describe the feasibility and effectiveness of an implementation model in a real-world setting, evaluating patient compliance with medical advice and adherence to a TEP. This intervention approach is based on providing individualized exercise prescriptions and supervised exercise sessions, while guiding patients through an educational pathway that promotes exercise as medicine, especially for those with multiple chronic conditions.

## Methods

### Design, setting and participants

In this real-world retrospective observational study, patients with various chronic diseases were consecutively assessed at the Sports and Exercise Medicine Division of the Department of Medicine, University of Padova (Italy) between 2014–2020, to initiate an exercise program. All subjects gave written informed consent in accordance with the Declaration of Helsinki, and the study was approved by the local Ethics Committee of the University Hospital of Padova (numbers 2892P, 43079 and 302n/AO/22).

**Fig. 1:**
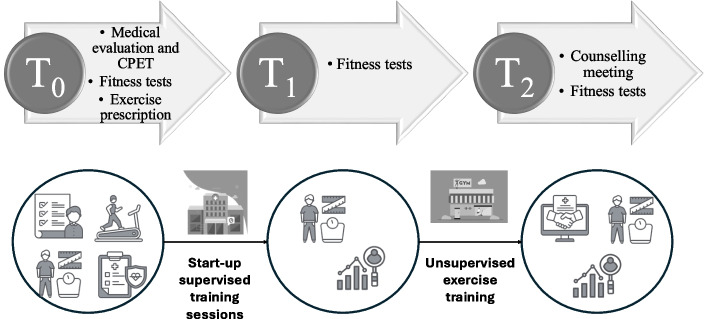
Study design and setting. Abbreviation: CPET, cardiopulmonary exercise testing

At baseline, patients were evaluated in an outpatient clinic with CPET and a functional fitness test battery (T0). Consequently, a TEP was provided for each participant by a Sports and Exercise Medicine Physician. Supervised training sessions (STS) were then performed in the hospital gym for at least 6 weeks, followed by a final functional evaluation with the fitness test battery (T1). Lastly, the TEP was adapted for a 6-month unsupervised out-of-hospital gym training, organizing the final follow-up meeting with the same functional evaluations (T2; Fig. 1).

Compliance was measured as the percentage of patients returning for the follow-up visit as recommended by the physician. Adherence to exercise therapy was measured by evaluating patients’ PA level during the 6-month of unsupervised training, focusing on frequency, type, and volume of exercise performed weekly using a structured interview. The feasibility of the intervention was assessed based on participants' ability to adhere to the exercise programs, the presence or absence of exercise-related adverse events, and the acceptability of the intervention, determined by the percentage of participants who completed the supervised training sessions.

### Procedures

#### Medical evaluation

At baseline, personal history was recorded and a physical examination conducted. PA levels were assessed through a standardized interview based on the physical activity vital sign questions, while also exploring leisure-time physical activities beyond the structured interview [24]. Patients were consequently divided into three groups: (a) physically inactive patients; (b) patients engaging in leisure time PA, such as walking with pets, gardening, etc.; (c) patients performing structured exercise training but not meeting international medical recommendations for their disease [24]. Anthropometric measurements such as height and weight were collected using a stadiometer and an electronic scale (Tanita, Arlington Height, IL, USA), respectively.

#### Cardiopulmonary exercise testing

At baseline, each patient performed an incremental maximal CPET, preferably on a treadmill (COSMOS, T170 DE-med model) with the modified Bruce protocol. Bicycle ergometer testing with individually adapted protocols was used for patients with orthopedic limitations or gait disturbances [25, 26]. A12-leads electrocardiogram, arterial blood pressure, and peripheral oxygen saturation were continuously monitored at rest, during exercise, and in the recovery phase. Ventilatory and gas exchange measurements were sampled breath-by-breath and measured by a low-resistance turbine and mass spectrometry, respectively (Masterscreen CPX Jaeger, Carefusion, Hoechberg, GE system) [4]. Criteria for exhaustion included a Borg rating of perceived exertion ≥ of 18/20, associated with either a maximal heart rate (HR) ≥ 85% of predicted (220 bpm – age) or a peak Respiratory Exchange Ratio (RER) > 1.10 [27, 28]. Patients were verbally encouraged to reach maximal exertion.

#### Fitness test battery

As a comprehensive evaluation of physical function, patients underwent the Senior Fitness Test at all assessments (T0-T1-T2). Specifically, muscular strength was evaluated with the Arm Curl Test and the 30-s Chair Sit-to-stand Test for the upper and lower body strength, respectively. Flexibility was bilaterally evaluated with the Back Scratch test and the Chair Sit and Reach test. Agility and dynamic balance were assessed with the Timed Up and Go Test. General procedures of these field tests have been largely described and validated elsewhere [29].

#### Tailored exercise prescription

TEP was performed by physicians using the “Frequency – Intensity – Type – Time – Volume – Progression” (FITT-VP) model as suggested by the American College of Sport Medicine guidelines [30]. Every TEP comprised four main parts:

1. Useful clinical information considering medical treatments that need to be taken into account for the TEP and its application.
2. Measurable clinical and functional goals of the exercise intervention shared with each patient.
3. A structured exercise prescription including endurance exercise, resistance training and adapted flexibility and balance training program following specific international guidelines. The patients’ main chronic conditions and comorbidities were considered for FITT-VP. The baseline PA level helped define the starting point and progression of exercise. Intensities for endurance training were set by HR at ventilatory thresholds measured during CPET. Intensities for resistance training were prescribed using the Rate of Perceived Exertion (RPE) scale, also considering the SFT results or submaximal repetition maximum (RM) test outcomes.
4. Lists of recommendations, specific precautionary measures and limitations due to specific comorbidities or risk factors associated with exercise training were also contextualized and discussed with the patients.

Psychosocial factors, potential facilitators and barriers to exercise were also considered based on the initial medical consultation, in order to further personalize the exercise prescription.

Finally, the TEP was provided as a written document containing all the aforementioned information and instructions, which was given to the patient at the start of the exercise initiation program.

#### Exercise training intervention

The exercise training intervention took place in an outpatient hospital gym. Exercise Professionals developed an individualized exercise program, based on the baseline functional assessments, the TEP, and the needs identified for each patient.

A first meeting in the hospital gym was organized to familiarize patients with the location, arrange exercise sessions, and conduct a counseling interview to set specific short-term goals. These goals included reconditioning, improved physical function, weight management, better disease control or risk factor reduction, as well as education on maintaining a physically active lifestyle, and the development of self-perception and body awareness.

Following this initial meeting, STS were conducted twice a week for 60–75 min each. Sessions started with breathing exercises, joint mobility warm-ups, and aerobic exercise training; HR was set and monitored according to the individual TEP. For aerobic training, a target heart rate range was provided based on the previously performed CPET. Participants started progressively with light intensities, monitoring treadmill speed and incline, or wattage and cadence (revolutions per minute) on a bike, to reach the prescribed intensity during the sessions and maintain it for the agreed duration. Subsequently, resistance, proprioception and flexibility training were performed, targeting all major muscle groups, using calisthenics or machines. Each exercise was initially performed for 1–2 sets, to achieve at the end three sets of 12–15 repetitions per set. When possible, submaximal test (10-Repetition Maximum) was also performed to determine the appropriate percentage of load for each machine used [28]. When this was not possible, intensity was based on the SFT results and the prescribed RPE, as mentioned above. An Exercise Professional supervised all sessions and always updated the program with stepwise progression as required. In addition, participants were encouraged to practice aerobic exercise sessions independently to increase their weekly PA volume. Intensity and exercise volume could vary during the program based on patients’ health conditions, with contraindications being excluded before each session.

After the supervised exercise initiation program, which lasted from 5 to 7 weeks, patients were encouraged to continue with regular exercise practice outside the hospital gym, adhering to the instructions provided in the initially delivered written exercise prescription, either by joining a local fitness center or training independently.

#### Follow-up

A 6-month follow-up was performed in the hospital gym by the Exercise Professionals. The fitness test battery was repeated to assess the effect of exercise training on physical function. Physical activity levels were also re-evaluated using the same structured interview as at baseline.

Additionally, another counseling session was conducted, in which long-term strategies were discussed with each patient to maintain an active lifestyle and pursue longer-term goals related to exercise adherence, such as improvement of physical efficiency, enhancement of quality of life, maintenance of regular exercise practice, and preservation of goals achieved in the short term. This was done in order to support patients in maintaining an active lifestyle beyond the study period.

A specific questionnaire was structured and administered by telephone calls, by a physician or an Exercise Professional, to not-compliant patients, i.e. those who did not attend the follow-up evaluation (See Additional file 1). The questionnaire explored their physical activity levels and habits during childhood/adolescence, reasons for potential inactivity, educational and marital status, occupation, as well as the specific reasons for not attending the 6-month follow-up and whether they continued exercising independently after the supervised training sessions.

### Data collection and statistical analyses

Statistical analyses were performed using SPSS (Version 21.0 for Windows, SPSS Inc., Chicago, IL). Results are expressed as mean ± standard deviation. Differences between data were assessed with t-test or non-parametric test depending on their distribution, assessed by Shapiro–Wilk test. Population characteristics were compared with the chi-square test or Fisher’s test depending on their numerousness. Statistical significance was defined as *p* < 0.05.

## Results

From 2014 to 2020, 312 patients with a mean age of 52.1 ± 13.6 years were enrolled.

Anthropometric measurements and clinical characteristics are described in Table 1. The most common chronic conditions were obesity (47%), solid organ transplantation (32%), primary cardiovascular diseases (chronic coronary artery disease, hypertensive cardiopathy, peripheral artery diseases; 8%) and cancer (4%). Patients’ functional test outcomes at the baseline evaluation are reported in Table 2.

**Table 1:** Anthropometric measurements and clinical characteristics of the whole sample

	Overall *N* = 312	Obesity *N* = 145	Solid organ transplantation *N* = 101	CVDs *N* = 27	Cancer *N* = 12	Other *N* = 27
*Age (years)*	52.1 ± 13.6	49.1 ± 12.5	52.8 ± 11.6	67.1 ± 11.7	49.1 ± 17.8	51.9 ± 16.1
*Sex (M/F)*	138/174	35/110	64/37	20/7	5/7	14/13
*BMI (kg/m*^*2*^*)*	30.7 ± 7.9	36.3 ± 7.4	25.7 ± 4.1	26.4 ± 3.2	25.1 ± 5.0	25.2 ± 4.7
PA level						
*Sedentary (%)*	56.3	63.4	48.5	40.7	75.0	53.8
*Leisure time PA (%)*	28.3	22.8	33.7	40.7	8.3	34.6
*Structured exercise (%)*	15.4	13.8	17.8	18.5	16.7	11.5
Comorbidities						
*Pre T2DM (%)*	4.7	8.5	1	16.7	0	0
*T2DM (%)*	18.5	16.2	20.8	4.2	10.0	28.6
*DLP (%)*	9.1	9.2	4	25.0	10.0	14.3
*HYPT (%)*	36.9	34.5	36.6	54.2	20.0	42.9
*OSA (%)*	6.0	9.9	2	4.2	0	4.8
*Osteoporosis (%)*	4.0	0.7	6.9	0	20.0	9.5
*Orthopedic disorders (%)*	41.6	45.8	35.6	25.0	30.0	66.7
*Psychiatric disorders (%)*	17.5	17.7	15.8	20.8	30.0	14.3

Data are expressed as mean ± standard deviation for continuous variables and as percentage for dichotomous variables

*Abbreviations*: *CVDs* Cardiovascular diseases, *M* Male, *F* Female, *BMI* Body mass index, *PA* Physical activity, *T2DM* Type 2 diabetes mellitus, *DLP* Dyslipidemia, *HYPT* Arterial hypertension, *OSA* Obstructive sleep apnea syndrome

**Table 2:** Functional evaluations at baseline

	Overall *N* = 312	Obesity *N* = 145	Solid organ transplantation *N* = 101	CVDs *N* = 27	Cancer *N* = 12	Other *N* = 27
*VO*_*2*_*peak (L/min)*	1.88 ± 0.58	2.10 ± 0.56	1.66 ± 0.54	1.76 ± 0.59	1.77 ± 0.38	1.62 ± 0.49
*VO*_*2*_*peak/kg (mL/Kg min)*	22.2 ± 5.7	21.5 ± 4.6	22.2 ± 6.1	23.1 ± 6.8	27.3 ± 5.9	24.1 ± 8.0
*VO*_*2*_*peak predicted (%)*	97.4 ± 24.5	108.3 ± 20.1	82.2 ± 20.1	100.1 ± 27.5	99.5 ± 24.2	92.7 ± 29.6
*OUES (L/logL)*	1859.2 ± 591.7	2080.6 ± 592.7	1632.5 ± 485.9	1794.9 ± 629.7	1696.2 ± 299.6	1527.8 ± 511.4
*VO*_*2*_* AT (mL/kg min)*	13.14 ± 3.3	13.4 ± 3.0	12.4 ± 3.0	12.6 ± 3.9	14.9 ± 5.8	13.8 ± 4.0
*VO*_*2*_* AT percentage of VO*_*2*_* peak (%)*	60.5 ± 11.2	63.1 ± 10.5	57.7 ± 11.5	58.3 ± 11.3	55.2 ± 9.1	58.3 ± 11.4
*HR AT (bpm)*	108.7 ± 19.2	115.8 ± 18.6	101.1 ± 16.1	94.4 ± 15.1	124.8 ± 16.6	101.8 ± 16.8
*HR AT percentage of HR max (%)*	74.2 ± 8.2	74.3 ± 8.1	74.6 ± 8.4	74.6 ± 8.0	72.8 ± 3.5	72.6 ± 9.3
*VO*_*2*_* RCP (mL/kg min)*	17.6 ± 4.6	17.4 ± 3.9	17.3 ± 4.6	16.6 ± 5.1	20.9 ± 6.3	20.1 ± 6.3
*VO*_*2*_* RCP percentage of VO*_*2*_* peak (%)*	79.7 ± 9.2	81.2 ± 8.5	78.5 ± 9.8	74.2 ± 9.1	79.2 ± 8.4	79.8 ± 9.8
*HR RCP (bpm)*	128.8 ± 23.3	136.7 ± 21.2	118.0 ± 21.3	111.2 ± 20.1	152.7 ± 23.7	126.8 ± 20.0
*HR RCP percentage of HR max (%)*	86.3 ± 6.2	86.8 ± 6.1	85.7 ± 6.3	84.7 ± 5.6	88.0 ± 1.9	86.2 ± 7.2
*RER peak*	1.2 ± 0.10	1.16 ± 0.10	1.18 ± 0.15	1.13 ± 0.10	1.28 ± 0.12	1.18 ± 0.12
*HR max (bpm)*	147.0 ± 26.0	155.1 ± 22.5	138.0 ± 26.2	129.9 ± 19.6	170.4 ± 22.6	142.8 ± 28.7
*HR max predicted (%)*	86.9 ± 13.2	90.1 ± 10.8	82.0 ± 14.7	84.3 ± 10.7	97.7 ± 8.5	85.7 ± 16.2
Fitness tests battery						
*30 SCT (n)*	10.8 ± 3.3	11.2 ± 3.5	10.2 ± 3.1	11.0 ± 2.4	11.2 ± 2.0	9.9 ± 4.1
*TUG (s)*	6.5 ± 1.7	6.4 ± 1.4	6.7 ± 1.8	6.2 ± 1.6	6.0 ± 0.6	7.0 ± 2.7
*AC right (n)*	17.4 ± 5.1	18.1 ± 4.7	17.1 ± 5.6	17.8 ± 4.8	15.3 ± 4.3	14.8 ± 4.6
*AC left (n)*	17.6 ± 4.9	18.3 ± 4.4	17.3 ± 5.6	17.1 ± 4.8	16.3 ± 4.5	15.1 ± 4.8
*S&R right (cm)*	(−6.1) ± (12.1)	(−4.4) ± (11.3)	(−6.3) ± (12.9)	(−11.9) ± (12.4)	(−0.5) ± (14.2)	(−12.4) ± (9.5)
*S&R left (cm)*	(−5.3) ± (11.9)	(−3.9) ± (11.4)	(−4.6) ± (12.0)	(−10.9) ± (11.6)	(−0.8) ± (13.0)	(−12.0) ± (10.6)
*BS right (cm)*	(−6.9) ± (11.0)	(−8.7) ± (10.6)	(−4.1) ± (10.7)	(−12.9) ± (12.1)	0.3 ± 4.6	(−4.9) ± (10.8)
*BS left (cm)*	(−10.5) ± (11.7)	(−11.8) ± (11.2)	(−8.3) ± (11.7)	(−14.6) ± (11.6)	(−5.2) ± (7.5)	(−10.6) ± (14.0)

Data are expressed as mean ± standard deviation for continuous variables and as percentage for dichotomous variables

*Abbreviations*: *CVDs* Cardiovascular diseases, *VO*_*2*_*peak* Peak of oxygen consumption, *VO*_*2*_*peak/kg* Weight-adjusted VO_2_peak, *OUES* Oxygen uptake efficiency slope, *HR* Heart rate, *AT* Anaerobic threshold, *RCP* Respiratory compensation point, *RER* Respiratory exchange ratio, *30 SCT* Chair Stand Test in 30 s, *TUG* Time up and go test, *AC* Arm curl test, *S&R* Sit and Reach test, *BS* Back Scratch Test

The subsequent initial program of STS was completed by 85.9% of patients and it lasted on average 11.03 ± 3 training sessions. Patients were able to adhere to the prescribed exercise programs without issues, as they were tailored to their individual needs and capabilities. No adverse events related to the prescribed exercise training intervention were reported during the STS program or at the follow-up evaluation.

The compliance at the 6-month follow-up meeting was 53.2%, without differences between patients with different chronic conditions. In those patients evaluated at follow-up, the adherence to prescribed exercise in the 6 months was 65.7% (44.9% considering the whole population).

A subgroup analysis was conducted to compare the baseline characteristics of patients depending on their compliance and adherence to the program (See Additional file 1_Table 1S). It was observed that compliant patients had a significantly higher basal VO_2_ peak compared to not-compliant patients (22.9 ± 6.1 mL/kg/min versus 21.4 ± 5.2 mL/kg/min, *p* = 0.023). The same trend was confirmed by observing adherent versus not-adherent patients (23.5 ± 6.3 mL/kg/min versus 21.5 ± 5.2 mL/kg/min, *p* = 0.003). On the other hand, starting PA level did not differ between groups. There were no significant differences between the groups regarding other anthropometric or clinical characteristics.

Not-compliant patients were affected by different chronic conditions: obesity 46.5%, solid organ transplantation 33.3%, or other chronic conditions 20.2%. Focusing on the administered questionnaire in this subgroup of patients only 32% reported regular PA in adolescence. The complete questionnaire results are shown in Additional files (See Additional file 2).

**Fig. 2:**
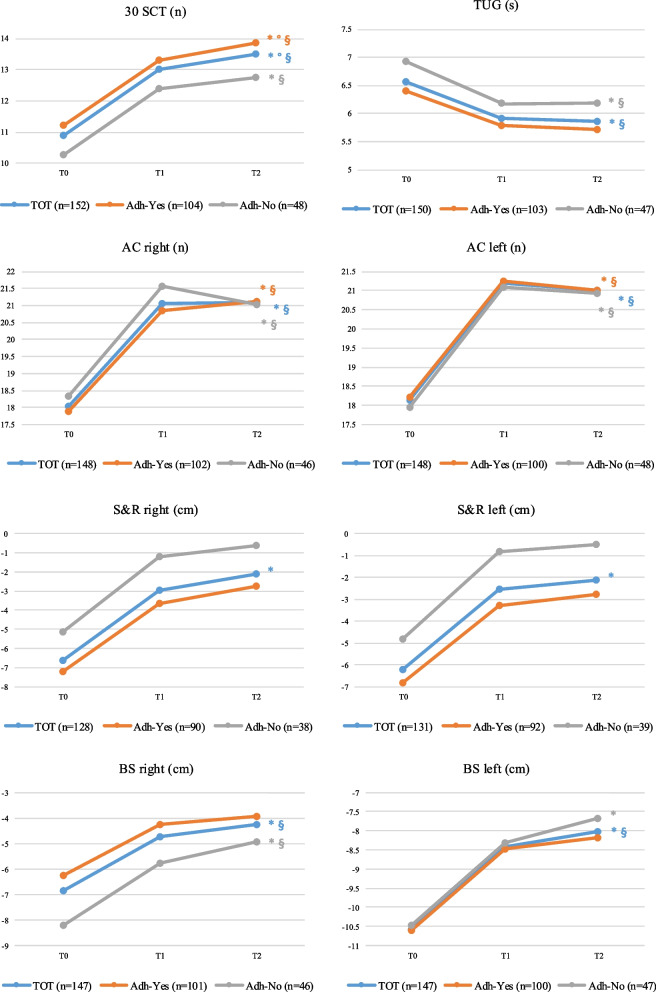
Effectiveness of the model in Fitness Tests, also evaluated in adherent and not-adherent patients. Fitness Tests Battery Results at baseline (T0), after Supervised Training Sessions (T1) and at mid-term follow up evaluation (T2). Difference between paired results in different time evaluation are shown as follows: *p<0.05 T0-T1; °*p*<0.05 T1-T2; §*p*<0.05 T0-T2. Abbreviations: 30 SCT, Chair Stand Test in 30 seconds; TUG, Time up and go test; AC, arm curl test; S&R, Sit and Reach test; BS, Back Scratch Test. Adh-Yes, adherent patients; Adh-No, not-adherent patients

Effectiveness of STS and subsequent exercise training was evaluated through fitness tests. Results of the tests battery at the three evaluations, also differentiated by adherence to the prescribed exercise therapy, are represented in Fig. 2. Improvement over time of adherent and not-adherent patients are described in Table 3.

**Table 3:** Percentage of improvement in adherent and not-adherent patients after the STS

	*Differences between T2 and T1 (%)* *Adh-Yes*	*Differences between T2 and T1 (%)* *Adh-No*	*p*
30 SCT	−0.08 ± 0.42	−0.39 ± 0.57	**< *0.0001***
TUG	−0.16 ± 0.36	−0.43 ± 0.51	**< *0.0001***
AC right	−0.08 ± 0.55	−0.42 ± 0.51	**< *0.0001***
AC left	−0.14 ± 0.41	−0.41 ± 0.51	**< *0.0001***
S&R right	−0.35 ± 1.45	−0.37 ± 1.18	0.903
S&R left	−0.02 ± 1.61	−0.57 ± 1.66	***0.027***
BS right	−0.06 ± 1.28	−0.42 ±.72	***0.010***
BS left	0.13 ± 1.73	−0.46 ± 0.8	***0.001***

*Abbreviations*: *30 SCT* Chair Stand Test in 30 s, *TUG* Time up and go test, *AC* Arm curl test, *S&R* Sit and Reach test, *BS* Back Scratch Test, *Adh-Yes* adherent patients, *Adh-No* Not-adherent patients

Among adherent patients, significant continuous improvements were observed from baseline to immediately after STS and to 6-month follow-up in the 30-Second Chair Stand Test (*p* < 0.0001) and the right side Arm Curl test (*p* < 0.0001). The left side Arm Curl test also significantly improved from baseline to post-STS (*p* < 0.0001) in this group.

Not-adherent patients experienced a significant decline from post-STS to 6-month follow-up in the bilateral Arm Curl test (right: *p* < 0.0001; left: *p* < 0.0001), as well as in the Timed Up and Go test (*p* < 0.0001).

Considering the overall sample, significant improvements were noted in all fitness tests from baseline to post-STS, which were maintained to 6-month follow-up for the 30-Second Chair Stand Test (*p* < 0.0001), the Timed Up and Go test (*p* < 0.0001) and the bilaterally Back Scratch test (*p* < 0.0001).

## Discussion

The present study aimed to evaluate the feasibility and effectiveness of an intervention approach to TEP in a real-world clinical healthcare setting for patients with chronic diseases, while also assessing compliance and adherence. We hypothesized that an initial STS program based on individualized exercise prescription could facilitate training interventions and promote mid-term adherence to a physically active lifestyle. Furthermore, our model was evaluated using an implementation science approach, focusing on a real world clinical setting [31, 32]. All exercise prescriptions were tailored for each patient following a comprehensive evaluation. Unlike clinical trials that typically involve selected patients and standardized approaches, our study reflects real-word scenarios, providing valuable insights into compliance and adherence to exercise prescriptions.

Our primary hypothesis regarding the feasibility and facilitation of the STS within a clinical pathway was supported, with 85.9% of patients completing the program. This completion rate aligns with the average of 79% observed in supervised exercise programs for various chronic diseases, including cardiac, pulmonary, and neurological conditions [33]. Additionally, our study support the safety of prescribing and administering adapted physical exercise for different chronic conditions in a real-word setting, as no adverse events occurred during functional evaluations or STS. These findings support the notion proposed by Pareja-Galeano et al. that exercise can be considered a “polypill” for treating almost every chronic disease, with benefits outweighing potential risks [10].

At baseline, patients exhibited an average low level of cardiorespiratory fitness (VO_2_ peak 22.2 ± 5.7 mL/Kg/min, mean age 52.1 years old, 44.2% male) compared to healthy subjects in the FRIEND registry [34]. This deconditioning was further confirmed by the 30-s chair stand test; with results comparable to the 50th percentile of individuals aged 80–89 years [35]. The significant deconditioning observed in these chronic patients likely stems also from their low baseline levels of PA, with 56.3% of participants being physically inactive and only 15.4% engaging in regular exercise without meeting international recommendations [29, 36].

Analyzing baseline clinical and functional characteristics, we divided patients into two groups based both on compliance and adherence. Compliant patients demonstrated higher functional capacity throughout the study period compared to not-compliant patients, with no significant differences in mean age and sex distribution. This trend was also observed in adherent versus not-adherent patients. Patients with better baseline fitness may be more intrinsically motivated to adhere to the training program, as they already experience the benefits of exercise on their health and well-being [37, 38]. One in five compliant patients already practiced structured exercise, although the starting total PA level did not significantly differ between groups. At the same time, greater confidence in their ability to perform exercise may also make these patients feel more comfortable and competent in following structured exercise programs, reducing intimidation barriers [39].

### Senior fitness test

All analyzed tests in the whole population showed improvement after the initial STS period, with maintenance in the mid-term. This suggests that initiating a structured training intervention with STS can provide lasting benefits for at least six months. The importance of adhering to exercise training over time is highlighted by the observation that not-adherent patients experienced a more significant decline in nearly all tests conducted. As is well known in the literature, after an initial period of improvement, maintenance of results is only achieved with constant exercise [40].

In the Arm Curl test and the Timed Up and Go test, we observed a linear improvement from T0 to T1 for the entire sample, with subsequent worsening only in the not-adherent group. This may be justified by the fact that, while other functional capacities can also be stimulated during activities of daily living, such as walking or general mobility, the arm curl strength and the dynamic balance could deteriorate if not regularly trained. These results underscore the importance of ongoing participation in structured exercise to prevent the decline of specific functional abilities like strength and agility [41].

### Compliance and adherence

Poor adherence to exercise programs has been identified as a major barrier to increasing physical activity levels [42]. When prescribing physical exercise, it is essential to address lifestyle habits in such patients. For this reason, real world scenarios must be observed beyond clinical trial settings, focusing on both compliance and adherence [8]. Our study applied supervised training methods previously described only in controlled trials and provided mid-term compliance and adherence data in a varied population. The mid-term adherence rate in our intervention is comparable to other studies including specific chronic diseases, such as cancer (65%), cardiovascular diseases (90%), and diabetes (80%), as reported in a systematic review and meta-analysis [43]. Nevertheless, among compliant patients in our sample, 34.3% no longer engaged in regular PA after the STS. This finding highlights an important issue: exercise adherence significantly declines after the termination of supervised programs, with many people ceasing altogether [41]. According to our telephone questionnaire administered to not-compliant patients, 53.7% continued exercising after STS but did not return for follow-up because they forgot, probably they did not think the meeting was very important. Considering long-term behavioral lifestyle change as the ultimate clinical goal, we can deduce that while short-term programs must focus on the safe, progressive and effective adoption of exercise training, as well as the development of self-perception and body awareness, long-term programs may require a different approach to maintain regular exercise behavior and foster an intrinsic change of mindset.

### Limitations and perspectives

Although this study boasts a good number of different patients from a local center, some limitations must be acknowledged. First, objective comparative measures at the 6-month follow-up evaluation between compliant and not-compliant patients were unavailable, as well as the questionnaire administered to the not-compliant group was not administered to compliant patients, limiting further insights. Secondly, measuring physical activity levels using brief questions can be considered restraint, as such self-assessment methods are generally less reliable than objective measurements. Finally, the feasibility assessment did not include a cost assessment; this aspect, however, could represent a fundamental obstacle to the implementation of this model.

Future prospective studies should consider more in-depth investigations among different patient groups to better clarify certain barriers that might led to drop-out, also providing comparisons with other implementation models. Furthermore, a recent umbrella review identified fourteen key factors as relevant for increasing exercise adherence, such as providing feedback, using mobile apps or wearable devices, promoting self-efficacy, social support, and enjoyment of the exercise activities [44]. In particular, the use of wearable devices would have allowed for more accurate monitoring of physical activity levels in the present study, which would helped motivate patients to remain active, as demonstrated in another study involving kidney transplant recipients [45].

Developing studies with external validation in real-world settings could help generalize results and provide valuable information. This would improve the implementation design of training interventions in healthcare settings, promoting exercise adherence in patients with various chronic diseases.

## Conclusion

In conclusion, a real-world implementation model involving medical evaluation, tailored exercise prescription, and supervised training sessions with Exercise Professionals is safe and effective in the short-term, also impacting mid-term follow-up outcomes.

Despite achieving a compliance rate of two out of three patients for a medical intervention program that includes exercise therapy, only half maintained adherence to exercise after 6 months.

Intervention strategies based on implementation science, behavioral change and motivation, as well as cost-effectiveness assessments, are needed to foster greater adherence to physical exercise in the mid-to-long term.

## Supplementary Information

Additional file 1: Table 1S.docx

Additional file 2: Questionnaire.docx

## Data Availability

The datasets used and/or analysed during the current study are available from the corresponding author on reasonable request.
