# Supplementary material for: From theory into practice: insights from a real-world implementation model for tailored exercise prescription in chronic diseases
Source: BMC Sports Sci Med Rehabil. 2025 Dec 29;17:373. doi: 10.1186/s13102-025-01419-5 (PMC12752253; doi:10.1186/s13102-025-01419-5)
Supplement: Supplementary file 1 — Additional file 1: Table 1S.docx [file 13102_2025_1419_MOESM1_ESM.docx]

***Supplemental Digital Content***

Table 1S. Baseline characteristics differences between adherent and not-adherent patients

|  | Compliant patients (N=166) | Not-compliant patients (N=146) | p | Adherent patients (N=109) | Not-adherent patients (N=203) | p |
| --- | --- | --- | --- | --- | --- | --- |
| *Age (years)* | 52.8±14.3 | 51.3±12.7 | 0.314 | 52.0±15.5 | 52.2±12.4 | 0.885 |
| *Sex (M/F)* | 82/84 | 56/90 | 0.053 | 50/59 | 88/115 | 0.720 |
| *BMI (kg/m^2^)* | 30.7±8.0 | 30.6±7.9 | 0.896 | 30.5±8.3 | 30.7±7.7 | 0.789 |
| *T2DM (%)* | 21.7 | 14.6 | 0.134 | 18.7 | 18.3 | 1 |
| *DLP (%)* | 8.7 | 9.5 | 0.842 | 7.5 | 9.9 | 0.534 |
| *HYPT (%)* | 40.4 | 32.8 | 0.111 | 36.4 | 37.2 | 1 |
| *OSA (%)* | 5.6 | 6.6 | 0.809 | 2.8 | 7.9 | 0.126 |
| *Osteoporosis (%)* | 4.3 | 3.6 | 1 | 3.7 | 4.2 | 1 |
| *Orthopedic disorders (%)* | 42.9 | 40.1 | 0.640 | 43.0 | 40.8 | 0.807 |
| *Psychiatric disorders (%)* | 16.9 | 18.2 | 0.762 | 16 | 18.3 | 0.750 |
| *VO_2_peak/kg (mL/Kg/min)* | 22.9±6.1 | 21.4±5.2 | **0.023** | 23.5±6.3 | 21.5±5.2 | **0.003** |
| *OUES (L/logL)* | 1906.6±571.0 | 1803.02±612.7 | 0.143 | 1910.3±582.1 | 1829.5±596.7 | 0.266 |
| *METs* | 8.9±3.4 | 9.0±3.2 | 0.770 | 9.2±3.6 | 8.8±3.1 | 0.305 |
| **PA level** |  |  |  |  |  |  |
| *Sedentary (%)* | 57.8 | 54.1 | 0.568 | 52.3 | 58.1 | 0.340 |
| *Leisure time activities (%)* | 23.5 | 33.6 | 0.059 | 27.5 | 28.6 | 0.895 |
| *Structured exercise (%)* | 18.1 | 12.3 | 0.208 | 20.2 | 12.8 | 0.100 |

M, male; F, female; BMI, body mass index; T2DM, Type 2 diabetes mellitus; DLP, dyslipidemia; HYPT, arterial hypertension; OSA, obstructive sleep apnea syndrome; PA, physical activity; VO_2_peak/kg, weigh-adjusted VO_2_peak; OUES, oxygen uptake efficiency slope; METs, Metabolic Equivalent of Tasks; AT, anaerobic threshold; RCP, respiratory compensation point; PA, physical activity.
