# Supplementary material for: From theory into practice: insights from a real-world implementation model for tailored exercise prescription in chronic diseases
Source: BMC Sports Sci Med Rehabil. 2025 Dec 29;17:373. doi: 10.1186/s13102-025-01419-5 (PMC12752253; doi:10.1186/s13102-025-01419-5)
Supplement: Supplementary file 2 — Additional file 2: Questionnaire.docx [file 13102_2025_1419_MOESM2_ESM.docx]

***Additional file***

Questionnaire administered by telephone to not-compliant patients and relative results (N=54).

1. During childhood/adolescence, did you practice structured physical exercise, in addition to school activities?

- No 48.1%
- Yes, occasionally 20.4%
- Yes, constantly 31.5%

1. If yes:

- for how many hours per week? 3.69 hours/week
- for how many years? 8.86 years
- Which type of activity? 82% competitive sports, 18% structured exercise in gym

1. If not, why did you not practice structured physical exercise?

- Lack of motivation/desire 20%
- Lack of time 4%
- Economic reasons 8%
- Familiar reasons 28%
- Healthy problems 16%
- Other… 24%

1. What is your educational qualification?

- Primary school degree 0%
- First degree secondary school degree 18.5%
- Second degree secondary school degree 55.6%
- Bachelor degree 25.9%

1. What was your marital status (during STS)?

- Maiden/celibate 33.3%
- Married 59.3%
- Widower 0%
- Divorced 7.4%

1. What was your job (during STS)?

- Freelance 24%
- Employee 53.7%
- Not working 3.7%
- Student 3.7%
- Retired 14.8%

1. Why didn’t you come back for a 6 months follow-up assessment at the hospital gym?

- I forgot 72.2%
- Healthy complications 9.3%
- Family problems 3.7%
- Troubles with my work 11.1%
- Lack of motivation 0%
- Other 1.9%

1. After STS, have you continued to do physical exercise?

- Yes, structured exercise training 53.7%
- No, but I maintained myself physically active 20.4%
- No, sedentary behavior 25.9%
